# An Account of the Successful Use of Opium, Cordial Drinks and Animal Food, in Two Cases of Pulmonary Consumption

**Published:** 1808-01

**Authors:** Benjamin Rush

**Affiliations:** Professor of Medicine in the University of Pennsylvania


*An Account of the successful Use of Opium, Cordial Drinks and Animal Food, in two Cases of Pulmonary Consumption.* By BENJAMIN RUSH, M. D. Professor of Medicine in the University of Pennsylvania.

ELIZABETH DAVIS, aged about twenty-five years, was admitted into the Pennsylvania hospital on the 7th of February, in apparently the last stage of pulmonary consumption, brought on by an attack of fever six months before. She expectorated pus in large quantities, had a diarrhœa, and spoke only in a whisper. She was moreover over so weak as to turn herself with difficulty in bed; her pulse had notwithstanding a small degree of tension. As I considered this tension in her pulse, an obstacle to the use of the only remedies that were indicated in her case, I directed four ounces of blood to be drawn from her, and ordered a blister to be applied to her breast, and small doses of calomel, tartarized antimony, and nitre, with a little laudanum, to be given to her every two hours. On the 12th of the month, I found her pulse so much changed into that state of weakness, softness, and frequency, that characterises a typhus fever, that I directed her to take two grains of opium every night. On the 19th she complained of a sore mouth, and of a difficulty in swallowing her opium pill: I now directed the calomel to be laid aside, and instead of the opium in a solid form, I ordered her to take eighty drops of laudanum at bed-time, and fifteen every morning and noon of every day.

On the 26th her diarrhœa returned, for which I prescribed the chalk julep, rendered cordial by an unusual quantity of laudanum. It had the desired effect. On the 1st of March I found her pulse evidently *slower* and *fuller* than it had been, and without any tension Her voice became stronger, her cough was less frequent, and her expectoration less copious than they had been, and she now complained only of pains in her head and back. I considered this change in her symptoms as highly favourable, and in order to secure the advantages thus obtained over her disease, I directed her to take 120 drops of laudanum and a pint of wine in the course of a day, and to live wholly upon the most cordial animal food. My tour of attendance at the hospital expiring at this time, I committed her to the care of my successor, Dr. Park, who politely continued the use of my prescriptions, by which means she was rapidly restored to health, and discharged cured on the 9th of April.

On the 6th of August, a woman with a full face and rosy countenance, came up to me as I was getting out of my chair to visit a patient in Fifth-street. She expressed her gratitude to me in strong terms for my services to her. I did not recollect her, and asked her name. With equal surprise and pleasure, I learned it was Elizabeth Davis. She appeared to be in perfect health.

Nicholas Messenger, a German, about thirty years of age, who had lately arrived in Philadelphia from his native country, was admitted into the Pennsylvania hospital on the 31st of October with a pulmonary consumption, brought brought on by a cold taken between two and three months before. All his symptoms, which were a severe cough, purulent expectoration, chills, fever, hoarseness, and a degree of weakness which confined him constantly to his bed, indicated a speedy and unfavourable issue of his disease. His pulse was too active for cordial remedies. I prescribed for him a blister to his breast, and the same mercurial and antimonial medicine with nitre, that I prescribed in the case of Elizabeth Davis. The mercury gently affected his mouth on the 14th of the month. His pulse felt its effects, and suddenly assumed the typhus action. I now directed the mercury to be discontinued, and ordered him to take two grains of opium every night, and a solution of liquorice in water with laudanum, to ease his cough during the day. I prescribed at the same time, drinks of the most cordial kind, particularly wine, and brandy toddy, and animal food of all kinds to be taken as often, and in as large quantities, as his stomach would bear them. On the 21st of the month, his pulse became *fuller* and *slower*. On the 23d, a diarrhœa, and a return of the weakness and frequency of his pulse induced me to order him to take three grains of opium at bed-time, and the chalk julep strongly impregnated with laudanum during the day. The complaint In his bowels soon yielded to these remedies. On the 1st of December his pulse again became *fuller* and *slower*. On the 5th he appeared to be much better. He slept soundly for three hours at night, coughed less, and ate his food with an appetite. On the 8th he left his bed, and walked out of his ward. On the 12th, his pulse became regular, and all the symptoms of disease left him, except a troublesome wakefulness, which often follows a recovery from violent and dangerous diseases. On the 15th he not only said he was well, but discovered the signs of a complete recovery in the return of flesh, a florid complexion, and in an ability to take exercise. On the 15th of January, 1805, he was discharged from the hospital, cured.

Both the above patients were seen during the whole course of my attendance upon them, by more than an hundred students of medicine. Upon these cases I shall make the following remarks.

1. It is difficult to tell the precise state of the lungs in these cases. It was evident a purulent expectoration took place in each of them. A similar issue from stimulating remedies should not be expected where the lungs are affected with tubercles. It is, however, in favour of those remedies, that consumptions with that issue from them, are less common in the United States than any others, more especially when they are of a recent nature.

2. The use of the stimulating remedies which have been mentioned, should be confined to a weak or typhus state of the pulse. In its inflammatory state they do harm, and in its hectic state they are generally ineffectual. It is not peculiar to the pulmonary consumption to resist the power of medicine, before it puts on its worst symptoms, or reaches its last stage. This was the case with the Jewish leprosy, and is still observed in several modern diseases.

3. It has been said, that there exists for every disease a specific remedy, and that sooner or later that remedy will be discovered. Without combating this assertion, I will say, that most diseases have a *precise* or *appropriate time*, in which only certain remedies do service. This remark applies with peculiar force to the pulmonary consumption, and it is because the remedies for this disease are administered without a due regard to the *different*, constantly *varying*, and often *opposite* states of the pulse, that bleeding, emetics, riding on horseback, a milk and vegetable diet, sea voyages, labour, digitalis, a salivation, opium, and stimulating drinks and diet, not only fail of success, but often do harm. The fable of the wax, which attempted to acquire the hardness of the brick, by throwing itself into the fire, is big with instructions to physicians under this head. We are less in want of new medicines, than of a knowledge of the *times* and *manner* of giving old ones, to enable us to cure obstinate and mortal diseases.

4. An increase in the fulness and a diminution of the frequency of the pulse, should always be considered as a signal to proceed with the use of stimulating remedies in this disease. An increase of the frequency of the pulse, or a revival of its tension from the use of those remedies, should always lead us to discontinue them.

5. Animal food was preferred in the above cases to bark and the common stimulants of the shops, upon the account of its furnishing the materials for a quick and plentiful supply of blood, and thereby producing fulness and force in the blood-vessels, (now nearly exhausted of their irritability) by its *direct* stimulus upon them, and thus enabling them to perform their healthy actions in every part of the body, and more especially in the lungs. Perhaps some advantage is derived from the stimulus of animal food upon the system by the pleasurable sensation it excites excites upon the tongue. This sensation is the more delightful, and the more diffused in its good effects, from the patients having been long deprived of animal food, and from their longing for it.

6. Lest an improper use should be made of the prescription of brandy toddy in the case of Nicholas Messenger, I shall conclude these remarks by adding, that I never prescribe ardent spirits as a cordial, except where I wish to obtain a *prompt* or immediate effect from them, in which case they are to be preferred to wine, and are often more acceptable to the stomach than opium. In chronic diseases of weak morbid action, and in simple debility without disease, ardent spirits cannot be taken long enough to produce a salutary effect, without endangering a subsequent attachment to them, and thus creating worse diseases than those they were taken to cure.

